# Diagnostic Criteria and Prognostic Relevance of Sarcopenia in Patients with Inflammatory Bowel Disease—A Systematic Review

**DOI:** 10.3390/jcm12144713

**Published:** 2023-07-16

**Authors:** Claudia-Gabriela Potcovaru, Petruța Violeta Filip, Oana-Maria Neagu, Laura Sorina Diaconu, Teodor Salmen, Delia Cinteză, Anca Pantea Stoian, Florin Bobirca, Mihai Berteanu, Corina Pop

**Affiliations:** 1 Doctoral School of “Carol Davila” University of Medicine and Pharmacy, 050474 Bucharest, Romania; claudia-gabriela.potcovaru@drd.umfcd.ro (C.-G.P.);; 2 Department of Gastroenterology and Internal Medicine, “Carol Davila” University of Medicine and Pharmacy, 050474 Bucharest, Romania; petruta.filip@umfcd.ro (P.V.F.);; 3 Department of Gastroenterology and Internal Medicine, Emergency University Hospital Bucharest, 050098 Bucharest, Romania; 4 Department of Physical and Rehabilitation Medicine, “Carol Davila” University of Medicine and Pharmacy, 050451 Bucharest, Romania; 5 Department of Diabetes, Nutrition and Metabolic Diseases, “Carol Davila” University of Medicine and Pharmacy, 050474 Bucharest, Romania; 6 Dr I. Cantacuzino Clinical Hospital General Surgery Discipline, “Carol Davila” University of Medicine and Pharmacy, 020021 Bucharest, Romania; 7 Department of Rehabilitation and Physical Medicine, University Emergency Hospital Elias, 011461 Bucharest, Romania

**Keywords:** sarcopenia, inflammatory bowel disease, malnutrition, frailty, quality of life

## Abstract

Background: Sarcopenia is a syndrome characteristic in elderly patients and is also associated with a significant proportion of chronic disorders such as inflammatory bowel disease (IBD). In this case, it can lead to a worse prognosis of the disease and a decreased quality of life. Study Aim: This study aims to identify the best ways to diagnose sarcopenia in patients with IBD, establish its impact on the course of the disease, and find preventive methods to counteract the effects of sarcopenia in the outcome of patients with IBD and, therefore, minimize disabilities and increase the health-related quality of life (HRQoL). Material and Methods: A systematic review with the Prospero registration number CRD42023398886 was performed in PubMed and Web of Science databases, evaluating all original articles published in the last 10 years (clinical trials and randomized control trials) that describe sarcopenia and IBD in the human adult population. Results: From the 16 articles that were included, 5 articles defined sarcopenia by the skeletal muscle index (SMI) and reported data regarding its correlation with body composition: BMI; visceral fat (VF); subcutaneous fat (SC); and VF/SC index. Other articles evaluated the link between sarcopenia and the total psoas muscle area, thigh circumference, calf circumference, subjective global assessment, hand grip strength, and appendicular SMI, alongside inflammatory markers such as IL-6 and C-reactive protein, level of disability, malnutrition, frailty, resistance training alone and in combination with whey protein, and infliximab treatment. Discussions and Conclusions: There is a great heterogeneity regarding the assessment criteria and methods used to diagnose sarcopenia due to the variability of population characteristics, both anthropometric and socio-cultural, alongside the high variability in the cut-offs. Therefore, any method which identifies sarcopenia in IBD patients, thus enabling intervention, may provide good results for patient quality of life and outcomes.

## 1. Introduction

Inflammatory bowel disease (IBD) has two main subtypes: Crohn’s disease (CD) and ulcerative colitis (UC), the characteristic of which is an immune-mediated chronic inflammation of the gastrointestinal tract, with episodes of remission and relapse [1]. Known as a disease in Westernized countries, the incidence of IBD has been rising in newly-industrialized countries in South America, Asia, and Africa. However, the highest reported prevalence of IBD remains in Europe and North America, with over 2 million and 1.5 million people diagnosed, respectively [2].

IBD can be associated with sarcopenia, which is usually a characteristic of the elderly and is known as primary sarcopenia. Secondary sarcopenia can be caused by insufficient activity, malnutrition, malignancy, congestive heart failure, chronic liver disease, chronic obstructive pulmonary disease, chronic inflammation, or steroid therapy. Sarcopenia is associated with physical frailty, limited mobility, increased risk of falls and fractures, and increased insulin resistance. It is, therefore, an important public health problem associated with disability and premature death. It is also considered to be a morbimortality risk factor [3,4]. There are two major types of muscle fiber: type I and type II, named slow-twitch and fast-twitch, respectively. Type I fibers are resistant to fatigue, generate little force, and are found more in elite endurance athletes. Type II fibers sustain short aerobic bursts of activity and are mainly found in elite strength and power athletes [5]. Sarcopenia is the term used to define the loss of skeletal muscle mass, which especially affects type II muscle fibers, together with muscle function and a progressive loss of motor neurons. Frailty is a dynamic syndrome found in the geriatric population that encompasses both physical and psychosocial factors, the definition of which overlaps with sarcopenia. However, it does not have a standardized diagnostic approach [6]. Frailty is associated with impaired quality of life (QoL), increased healthcare costs, healthcare use (prolonged hospitalization and skilled nursing facilities), and high mortality [7].

Studies reported an incidence of sarcopenia in IBD patients that ranged from 36.7% to 65% and concluded that sarcopenia has a negative impact on the length of hospital stay, surgical outcomes, clinical course, and quicker biologic agent failure of IBD patients [8,9]. Along with its manifestations, complications, and related affections, including sarcopenia, IBD can lead to disability, impaired functioning, and implicitly, a decrease in health-related quality of life (HRQoL) [10].

Since 2001, the novel approach of the World Health Organization (WHO) regarding disability has moved from describing disability exclusively from the viewpoint of health professionals towards a biopsychosocial model. This new paradigm is defined by the International Classification of Functioning, Disability, and Health (ICF) [11,12]. According to the ICF, disability is seen as the human experience of impaired body functions and structure, activity limitations, and participation restriction in interaction with environmental factors. The WHO Disability Assessment Schedule 2.0 (WHODAS 2.0), a tool that measures disability according to the ICF definition, shows that patients with IBD have greater restrictions regarding interpersonal relationships, life activities, and social participation [13]. Therefore, with an estimated annual total cost of 3.1–4.5 billion USD in the United States, IBD impacts the healthcare system in both direct and indirect ways [14].

**Figure 1:**
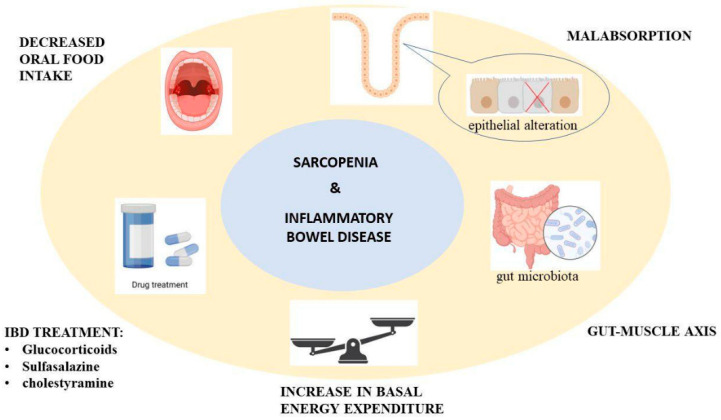
The proposed mechanism for sarcopenia through inflammatory bowel disease.

There are many mechanisms through which IBD causes sarcopenia. One of them is malnutrition, which accompanies IBD and is mainly caused by decreasing oral food intake and malabsorption, secondary to the epithelial alteration of the gut mucosae with altered transport functions. Impaired nutritional status is also caused by increased basal energy expenditure and the drugs used for treating IBD, like glucocorticoids, sulfasalazine, and cholestyramine [15]. The gut–muscle axis is the name given to the relationship between gut microbiota and sarcopenia. More studies are needed regarding this topic, but it is thought that muscle mass and function are affected by the capacity of gut microbiota to regulate systemic inflammation, immunity, energy metabolism, and insulin sensitivity, as seen in Figure 1 [16].

There are two important interventions for sarcopenia: nutritional supplementation and resistance training [17,18]. Resistance exercise promotes muscle mass gain and strength. There are studies that confirm that resistance exercise is better to counteract sarcopenia when using protein supplementation, essential amino acids, vitamin D, bisphosphonates, calcifediol, and calcium [19].

The aim of this study is to carry out a systematic review that addresses the diagnostic criteria used to define sarcopenia in patients with IBD to establish the prognostic relevance on the course of the disease and secondary to find preventive methods to counteract the effects of sarcopenia so as to decrease disability and increase HRQoL.

## 2. Materials and Methods

We registered a systematic review protocol under the number CRD42023398886 in Prospero that followed the recommendations of the Preferred Reporting Items for Systematic Reviews and Meta-Analyses (PRISMA).

### 2.1. Research Question and Search Strategy

The literature search was conducted using the electronic PubMed and Web of Science databases, following the keywords: (“sarcopenia diagnosis” [All Fields]) AND (“inflammatory bowel disease” [All Fields]), and 93 articles were identified (73 articles on PubMed and 20 on Web of Science), published in English, before 11 March 2023 as original articles. The research question was constructed according to the Population, Intervention, Comparison, and Outcome (PICO) method, and the population was represented by IBD adults’ population with a diagnosis of sarcopenia.

### 2.2. Inclusion Criteria

To be included in this review, studies had to meet the following publication criteria: (i) original full-text articles with randomized control and clinical trials; (ii) articles from the last ten years; (iii) articles published in English.

### 2.3. Exclusion Criteria

Studies were excluded from the analysis if (i) they involved patients with sarcopenia but no diagnosis of IBD; (ii) if the patients were children; (iii) were literature reviews. Literature reviews, meta-analyses, case reports, and abstracts were excluded from the selection, but they were used for additional references.

### 2.4. Selection of Studies

**Figure 2:**
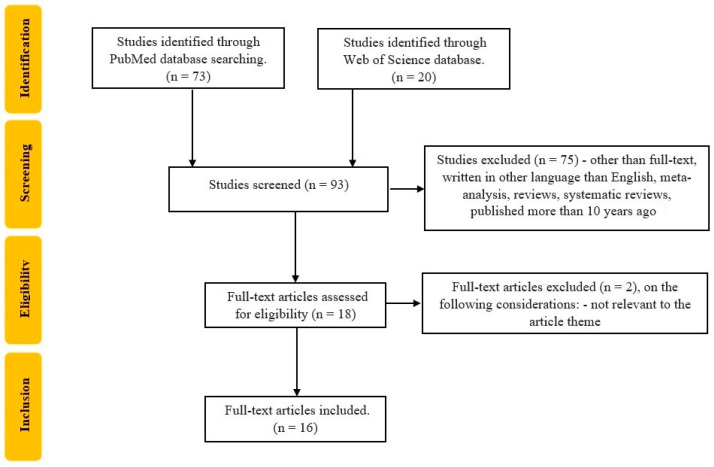
Flowchart of the study selection process according to PRISMA recommendations.

The selection of the studies was carried out by three reviewers (CGP, TS, PVF). Studies that were excluded from the analysis were articles that were not randomized control trials or original trials published in languages other than English. All data were independently recorded by CGP and TS in separate databases and only compared at the end of the reviewing process to limit the selection bias. PVF resolved any disagreements that appeared, as seen in Figure 2. Each reviewer read the identified papers to ensure that all predefined criteria were met and extracted the following data: title and study details (first author, year of the study); study population characteristics (total number, CD patients’ number, UC patients’ number); the methods that were used to assess skeletal muscle mass and strength.

## 3. Results

### Included Studies

The demographic characteristics of the patients that were included are presented in Table 1. Most studies were prospective cohort studies, and two were randomized control trials. As for the countries surveyed, we obtained ten from Asia, three from Europe, two from Oceania, and one from North America. The sample size ranged from 19 to 11,001 patients with IBD. The mean age of patients ranged from 32.5 to 46 years, while the BMI ranged from 18.49 to 24 kg/m^2^. One study did not report the characteristics of the entire population studied. It reported the characteristics only in subgroups of CD and UC patients, respectively.

From a total of 16 articles, 5 articles defined sarcopenia with the aid of the skeletal muscle index (SMI) with different cut-off values [28,29,31,34,35], as seen in Table 2.

Data on sarcopenia and its correlation with body composition—BMI, visceral fat (VF), subcutaneous fat (SC)—and VF/SC index, are seen in Table 3.

One article defined sarcopenia with the help of total psoas muscle area (TPA) divided by square of the height, with cut-off values <545 mm^2^/m^2^ for males and <385 mm^2^/m^2^ for females [23], one article considered that sarcopenia in CD patients was induced by mediators of systemic inflammation and assessed the impact of IFX on muscle volume and muscle strength in correlation with decreasing inflammatory markers IL-6 and C-reactive protein (CRP) [20]. Five articles assessed the effects of malnutrition on IBD patients and correlated it with sarcopenia and frailty [24,26,27,30,32]. One article used AWGS 2019 criteria as the reference standard in diagnosing sarcopenia. It aimed to explore cut-off point and diagnostic accuracy of thigh circumference (TC), calf circumference (CC), subjective global assessment (SGA), and hand grip strength (HGS) to identify sarcopenia [33]. One article defined sarcopenia using the appendicular skeletal muscle index (ASMI) as being less than two standard deviations below a young adult’s mean measured by whole-body dual-energy X-ray absorptiometry [21]. Two articles defined sarcopenia using AWGS 2019: one correlated sarcopenia with disability, and the second evaluated the effects of resistance training (RT) alone and in combination with whey protein (WP) on body composition and different blood markers [22,25].

Cocîrlan et al. [24] brought attention to the issue that malnutrition is an important predictor of poor prognosis in hospitalized IBD patients and that sarcopenia is more common in patients with a lower median BMI than patients with a normal range, 20–24.9 kg/m^2^.Boparai also concluded that sarcopenia has a negative influence on the outcomes (surgery and disease course) of CD. There were two studies that assessed some methods used to counteract muscle wasting in patients with IBD. Subramaniam et al. [20] concluded that IFX increases muscle volume and muscle strength independent of physical activity or diet. Zhao et al. [22] investigated the effect of nutritional supplementation and resistance training in managing sarcopenia. They concluded that resistance training and dietary supplements both improve ASM/H^2^, but they work best together. Table 4 synthesizes the tools and interventions used for the assessment of muscle gain, strength, and physical activity.

Bian et al. [25] assessed disability in patients with Crohn’s disease using the Inflammatory Bowel Disease Disability Index (IBD-DI) and saw a significant correlation between the grade of disability and ASMI, HGS, BMI, SGA, and CRP. A higher prevalence of sarcopenia and a higher Crohn’s disease activity index (CDAI) are related to moderate to severe disability.

## 4. Discussion

### 4.1. Assessment of Sarcopenia

Muscle mass reduction in sarcopenia is assessed through several radiological imaging-based techniques. In the case of CT, the most used cut-off values for muscle mass assessment range from 52 to 55 cm^2^/m^2^ for males and 39 to 41 cm^2^/m^2^ for females, in the condition of a threshold of muscle tissue of −29/+150 Hounsfield Units (HU) [36].

In the included articles in our study, the cut-off values ranged from 36.54 cm^2^/m^2^ in Boparai et al. to 52.4 cm^2^/m^2^ in Nardone et al. in the case of males and from 30.2 cm^2^/m^2^ in Boparai et al. to 38.5 cm^2^/m^2^ in Nardone et al. in the case of females. One of the possible explanations for this wide range variation in the cut-off values for SMI may be due to the nonuniform population included in the studies because, in Caucasian populations, there is a reported value of 52.8 ± 7.4 cm^2^/m^2^ for males and 40.2 ± 5.2 cm^2^/m^2^ in females. In comparison, in the Asian population, there is a reported value of 52.4 cm^2^/m^2^ for males and 41 cm^2^/m^2^ for females [37,38].

Boparai et al. [28] reported that the BMI in CD patients with sarcopenia was lower than in CD patients without sarcopenia, 17.2 ± 4.5 vs. 21.5 ± 3.4, *p* = 0.001, and so did Nardone et al., 20.3 ± 3.1 vs. 23.3 ± 3.8, *p* = 0.002, and Ge X et al., 20.2 ± 3.5 vs. 21.3 ± 3.0, *p*= 0.006 in UC patients, while Nam et al. reported that BMI is higher in both CD and UC patients with sarcopenia than in patients without sarcopenia, 20.1 ± 3.3 vs. 19.2 ± 3.3 *p* ˂ 0.0001, 22.4 ± 3.9 vs. 21.6 ± 2.7, *p* ˂ 0.0001, with no significant difference between CD and UC in the mean BMI [28,29,34,35,39]. Zhou et al. reported that low BMI is associated with adverse outcomes such as the need for intestinal surgery, initiation of anti-TNF therapy, or an escalation of biologic therapy. Jiang et al. reported that a BMI ≥ 30 kg/m^2^ is associated with more complications regarding surgery treatment, but Ding et al. reported that obesity is not associated with non-response or loss of response to anti-TNF [31,40,41]. Because BMI does not always reflect the body composition and there is a group of approximately 16% of CD patients with sarcopenic obesity, a personalized evaluation through other body composition tools, like VF, SC, and VF/SC ratio, should be used along with BMI for a better comprehension of sarcopenia [42].

Fat tissue can also be measured with the aid of CT with a threshold that ranges from −150 to −50 HU [39]. Boparai et al. reported that VF and SC areas were lower in CD patients with sarcopenia than in non-sarcopenic patients, 77.27 ± 36.9 vs. 109.2 ± 58.3, *p* = 0.04; 89.76 ± 65.1 vs. 141.7 ± 85.9, *p* = 0.03. Although the VF/SC ratio did not change between subgroups, it was considerably higher in individuals who received surgery compared to those who did not (1.76 ± 1.31 vs. 0.9 ± 0.41, *p* = 0.002), with a cut-off value for the VF/SC ratio of 0.88 in predicting surgery, with an area under the curve of 0.73 (0.52–0.95), sensitivity (Sen) of 71%, and specificity (Spe) of 65% [28]. Nardone et al. found that there was no difference in CD patients regarding VF area and the presence or absence of sarcopenia 54.0 ± 63.1 vs. 63.4 ± 62.7, *p* = 0.54, but found a lower SC area in patients with sarcopenia compared to non-sarcopenic patients, 94.1 ± 90.4 vs. 149.9 ± 84.2, *p* = 0.009, while the VF/SC ratio was higher in patients with sarcopenia compared with patients without sarcopenia 0.7 ± 0.6 vs. 0.4 ± 0.4, *p* = 0.04. Ge X et al. found that in UC patients, there was no correlation between sarcopenia status and VF area, 62.8 ± 40.3 vs. 66.4 ± 37.2, *p* = 0.457. However, for the SC area, there is a significant correlation, 96.2 ± 46.6 vs. 115.9 ± 50.4 *p* ˂ 0.0001. Furthermore, the VF/SC ratio was higher in patients with sarcopenia than those without sarcopenia, 0.75 ± 0.49 vs. 0.61 ± 0.31, *p* = 0.006 [29]. Nam et al. reported that the VF and SC areas were lower in sarcopenic patients than in non-sarcopenic patients for both CD and UC patients, 34.4 ± 28.4 vs. 49.7 ± 45.0, *p* ˂ 0.0001, 74.0 ± 57.2 vs. 93.4 ± 70.9, *p* ˂ 0.0001; 31.6 ± 27.2 vs. 42.4 ± 36.8, *p* = 0.032, 63.5 ± 45.6 vs. 83.2 ± 57.7, *p* = 0.015. Zhou et al. found that the adverse outcome rate was lower for patients with a lower VF/SC ratio than in patients without adverse outcomes (8.06 ± 25.98 vs. 1.22 ± 0.99, *p* = 0.026) [31]. Singh et al. reported that in IBD, there were significantly lower nutritional parameter values, such as mean BMI, mid-upper arm circumference (MUAC), triceps-fold thickness (TSF), mean fat and lean mass, as compared to controls. Moreover, in terms of sarcopenia, the hand-grip test and mean L3 SMI were lower in patients with IBD. To define malnutrition, a threshold value for fat mass (15.8 kg), fat mass index (FMI) (6.39 kg/m^2^), and visceral fat index (VFI) (0.16) had a Se ≥ 0.8. To detect malnutrition, the VFI (0.26) and free fat mass index (FFMI) (17.13 kg/m^2^) had a Se = 0.69 and Spe = 0.98 for females and Se = 0.7 and Spe = 1 for males. Among anthropometric measures, the threshold values for MUAC and TSF thickness were 23.25 and 25.25 cm, and 16.50 and 8.50 mm in females and males, respectively [43]. When talking about complications associated with IBD, Bamba et al. reported that the VF/SC index is associated with a prolonged length of hospitalization (≥30 days) 95% CI 1.005 (0.987–1.024), *p* = 0.561; 95% CI 1.231 (0.842–1.795), *p* = 0.038 for CD and 95% CI 1.002 (0.983–1.022), *p* = 0.793 for UC, respectively; intestinal resection 95% CI 1.039 (1.023–1.054), *p* < 0.001 for CD and 95% CI 1.046 (1.024–1.072), *p* < 0.001 for UC [44].

Holt et al. [21] defined sarcopenia by comparing ASMI to DXA and considered that an ASMI of less than two standard deviations below a young adult’s mean is consistent with sarcopenia. They observed that skeletal muscle area did not predict endoscopic outcomes, but calprotectin is lower when the ASMI is higher and concluded that patients with sarcopenia and CD have a mean baseline fecal calprotectin higher than patients without sarcopenia and CD (2570 ± 879 µg/g vs. 1095 ± 1074 µg/g, *p* = 0.003) [21].

The European Working Group on Sarcopenia in Older People (EWGSOP) proposed a diagnostic algorithm for sarcopenia in 2010 that included both muscle mass and muscle function and reviewed it in 2018. However, in 2014, the Asian Working Group for Sarcopenia (AWGS) proposed a different algorithm, which was reviewed in 2019, because there are different anthropometric (body size, higher adiposity) and cultural lifestyles (high activity level) between Asian and Western populations [45,46]. Three articles, all with an Asian population, defined sarcopenia using the AWGS 2019 criteria [22,25,33]. Bian et al. used bioelectrical impedance analysis (BIA) and HGS for assessing body composition and defined loss of skeletal muscle mass as an ASMI ˂ 7.0 kg/m^2^ for males and ˂5.7 kg/m^2^ for females and an HGS ˂ 28 kg force for males and ˂18 kg force for females. Pribadi et al. used dual-energy X-ray absorptiometry (DXA) for assessing body composition with the same parameters, and HGS ˂ 26 kg force for females and the same parameter for males. He assessed physical performance using a five-times chair stand test (5CST), with a 5CST ≥ 12 s for both males and females. There are few data regarding the evaluation of sarcopenia in IBD patients using the EWGSOP definition, and more research is needed. Using AWGS 2019 as a standard diagnostic technique, Pribadi et al. explored the cut-off point and diagnostic accuracy of CC, TC, and SGA to explore other inexpensive and simple examination techniques for assessing sarcopenia. He found that a CC of 31 cm is optimal and has a Sen, Spe, positive predictive value (PPV), negative predictive value (NPV) of 100%, 60.38%, 25%, 100%, a TC value of 50 cm is optimal with a Sen, Spe, PPV, NPV of 100%, 83.02%, 43.75%, 100% for identifying sarcopenia, and SGA, has a Sen, Spe, PPV, NPV of 42.86%, 84.91%, 27.27%, 91.84% [33]. A study investigated the diagnostic performance of sarcopenia in patients ˃60 years, with the combination of the CC, TC, and SARC-F questionnaire compared to the AWGS-2019 as being considered the standard diagnostic. They used a cut-off value of CC ˂34 cm in males and ˂29 cm in females, respectively, ˂49 cm in males and ˂44 in females for TC, and a SARC-F score ≥4. The AUC value of this combination was 86.2% (95% CI 0.76–0.96, *p* ˂ 0.001) [47].

Kang et al. [23] defined sarcopenia using TPA measured by total psoas muscle areas around the third vertebrae on computed tomography (CT) with cut-off values < 545 mm^2^/m^2^ for males and <385 mm^2^/m^2^ for females [23]. They assessed the link between sarcopenia and the risk of non-alcoholic fatty liver disease (NAFLD). They found that sarcopenia is more common in NAFLD patients compared to the non-NAFLD group (51.0% vs. 33.0%) and that it is a persistently independent risk factor for NAFLD in IBD patients (OR = 2.99; 95% CI 1.49–6.00; *p* = 0.002) and was maintained after adjustment for age, gender, obesity, diabetes mellitus, high blood pressure, and hyperuricemia (OR = 2.30; 95% CI 1.20–4.41; *p* = 0.012) and remained, after further adjustment for TG, HDL, and CRP (OR = 2.30; 95% CI 1.17–4.53; *p* = 0.016), and adjustment for small bowel resection history (OR = 2.23; 95% CI 1.13–4.41; *p* = 0.022) [23].

There are studies that indicate that when sarcopenia is correlated with NAFLD when the BMI is higher and correlates NAFLD with sarcopenic obesity [48]. The patients with lower muscle mass have a higher risk of developing NAFLD (OR = 3.42, 95% CI 1.30–8.96, *p* = 0.002) [49]. The link between metabolic syndrome and NAFLD is well known, but patients with IBD are usually young and have a lower risk of metabolic syndrome due to malabsorption, malnutrition, and the disease itself [50,51,52]. Lower SMI is associated with a higher fatty liver index (FLI): 43.2 ± 3.9 at an FLI ˂ 20, 40.2 ± 4.0 at an FLI between 20 and 59, and 38.2 ± 4.4 at an FLI ≥ 60. More studies are required to define NAFLD and its relationship with IBD patients with sarcopenia [53].

### 4.2. Interventions for Sarcopenia

Zhao et al. [22] applied two interventions on IBD patients diagnosed with sarcopenia with AWGS 2019 criteria: resistance training (RT) and whey protein (WP) and RT with a placebo and measured 5CST, 3-metre walking speed, BMI, waist-to-hip ratio, height-adjusted appendicular skeletal muscle mass (ASM/H^2^), using BIA, calf–waist–hip circumference, and GS in order to observe the effects of nutritional supplementation and RT on sarcopenia, and secondary, the effects on hemoglobin, creatinine, erythrocyte sedimentation rate (ESR), CRP, and albumin [22]. They concluded that at 4 weeks of intervention in the group with RT and WP, ASM/H^2^ was significantly higher than baseline (*p* = 0.035) and, therefore, CC, albumin, hemoglobin, and creatinine. Group RT + WP had higher scores than RT + placebo, and there was no significant group × time interaction for BMI (*p* = 0.065), 5CST (*p* = 0.309), 3-metre walk (*p* = 0.382), or GS (*p* = 0.059). It is well known that RT significantly improves SMI, GS, and gait speed in elderly patients with sarcopenia and that WP increases the ASM independent of the presence of a simultaneous physical activity program [54,55]. This is the first study that investigates the effects of RT and nutritional supplementation on IBD patients with sarcopenia, and the results should be replicated on larger lots of patients [22].

Subramaniam et al. [20] demonstrated that treatment with IFX in CD patients can produce a gain in muscle volume and muscle strength, independent of physical activity and dietary intake. The volume of quadriceps femoris was measured using MRI at the anatomical mid-thigh at three different times: 1, 16, and 25 weeks following IFX treatment. The muscle volumes were 1505 cm^3^ to 1607 cm^3^ (*p* = 0.012) and 1569 cm^3^ (*p* = 0.010) at the right quadriceps and 1478 cm^3^ to 1565 (*p* = 0.042) cm^3^ and 1534 cm^3^ (*p* = 0.010) in the left quadriceps femoris. Muscle strength (F) was measured at three speeds of contraction at 16 and 25 weeks after IFX treatment, with higher results after 25 weeks: 166.5 Nm at 30°/s to 199.5 Nm at 30°/s (*p* = 0.006), 172.8 Nm at 60°/s to 210.3 Nm *p* = 0.002 128.5 Nm at 90°/s to 155.5 Nm at 90°/s (*p* = 0.004). Along with the improvement in muscle volume and F, IFX decreased the CRP level from 31.4 mg/L at week 1 to 4.8 mg/L at week 16 (*p* = 0.001), and IL-6 levels from 4.195 ng/mL at week 1 to 0.175 ng/mL at week 25 (*p* = 0.037) [20].

In a narrative overview of sarcopenia in IBD patients, Dhaliwal et al. said that nutritional, physical, and pharmacological interventions were used successfully in the management of sarcopenia associated with IBD. Nutritional supplementation is beneficial for increasing muscle mass and strength, like a high dietary protein intake (especially amino acids) of 1.2–1.5 g/kg/day, which are often indicated during active disease states in an effort to stop muscle wasting, together with vitamin D and Omega 3 Polyunsaturated Fatty Acids supplementation. Physical activity, both aerobic and RT, is used for muscle mass and muscle strength improvement and also for anti-inflammatory effects. Prehabilitation (nutritional, exercise, and psychological treatment before a specific intervention) is known to shorten hospital stays and increase the percentage of colorectal surgery patients who live without disease for five years. This idea has the potential to have a significant impact on patients with IBD who are waiting for elective surgery. More studies to establish the need for prehabilitation for IDB patients who are in need of surgery are needed [56].

Data reported regarding the presence of sarcopenia at the diagnostic of CD, and their power to predict the need for surgery, are heterogeneous. Lee et al. found that sarcopenia was present in 50% of patients newly diagnosed with CD but that it is not a predicting factor for the need for surgery, initiation of steroids, immunomodulators, biologics, or hospitalization, while Ryan found that sarcopenia can predict the need for surgical intervention in IBD patients. It was associated with an increased rate of major postoperative complications, but that nutritional management may diminish this risk [57]. To enhance the prognosis of IBD patients, it is crucial to research potential therapies for sarcopenia in IBD patients, and there is a need for multidisciplinary teams: gastroenterologists to give the appropriate treatment to obtain the remission, physical rehabilitation physicians in order to prescribe a personalized set of exercises to fit best the needs of the patients and their associated pathologies; a diabetologist to give the appropriate nutritional advice; and a surgeon, if required, in the evolution of the disease.

Ultrasound (US) is a precise, repeatable technology for measuring muscle mass in various populations. Muscle characteristics appreciated by US are strongly correlated with muscle assessment by CT, MRI, or DXA [58,59]. The assessment of muscle mass using US is not standardized yet [60]. There is no study that assesses sarcopenia in IBD patients using US.

### 4.3. Malnutrition, Frailty, and Disability

Five studies assessed the relationship between malnutrition and IBD and found that malnourished patients were considered susceptible to developing sarcopenia. In older people, the association between sarcopenia and malnutrition was considered as a diagnosis of frailty, and sarcopenic patients were more likely to have a disability than patients without sarcopenia [25,26,27,30,32].

Fiorindi et al. [30] used the Global Leadership Initiative on Malnutrition (GLIM) criteria to assess malnutrition together with other malnutrition assessment tools like Nutritional Risk Screening (NRS) 2002 in IBD patients requiring surgical treatment, while Huang et al. used GLIM criteria and NRS to assess malnutrition in non-surgical IBD patients [27,30]. Huang et al. reported that 65.75% of patients had a nutritional risk factor according to NRS-2002 criteria, but 58.90% were malnourished according to the GLIM criteria. Based on GLIM classification, they reported that 28.77% of IBD patients had mild to moderate malnutrition. In comparison, 30.14% had severe malnutrition, with a higher rate of severe malnutrition in CD patients than in UC patients (35.42% vs. 20.00%) [27]. Fiorindi et al. reported a malnutrition prevalence of 42% according to GLIM criteria (15% stage 1 and 27% stage 2) with a proportion of 34% and 60% in CD and UC patients, respectively, with a higher prevalence of fistulizing disease behaviour in malnourished CD patients compared with non-malnourished patients (*p* = 0.01999) [30]. It is crucial to have an accurate approach to malnutrition identification since it is a predictor of postoperative complications in IBD patients with sarcopenia.

Fiorindi et al. [30] used GLIM criteria together with the European Society for Clinical Nutrition and Metabolism criteria (ESPEN 2015) and several malnutrition risk tools, such as NRS-2002, the Malnutrition Universal Screening Tool (MUST), the Malnutrition Screening Tool (MST), with two other tests specifically designed for the IBD population, the Malnutrition Inflammation Risk Tool (MIRT) and the Saskatchewan IBD–Nutrition Risk (SaskIBD-NR), to assess malnutrition in IBD patients before elective surgery treatment. The Kappa coefficient (k = 0.672) showed a moderate-to-good concordance between GLIM and ESPEN 2015 diagnosis of malnutrition. Independent GLIM variables, like FFMI and Non-volitional Weight Loss (NWL), are lower in patients who had undergone previous surgery than patients at first surgery (*p* = 0.017 and *p* = 0.041) and in patients with ileostomy (*p* = 0.03 and *p* = 0.002). According to GLIM criteria, NRS-2002 and MIRT, had the highest percentage of malnutrition, both with 40%, while SaskIBD-NR had the lowest detection level of malnutrition, respectively, 25%. NRS-2002 and MIRT had the lowest false negatives (*n* = 4), while SaskIBD-NR had the highest number of false negatives (*n* = 11) when GLIM was used to diagnose malnutrition [30]. So, ESPEN did not perform well in identifying patients with malnutrition, but it is a good prognostic tool among hospitalized patients, especially when used in combination with NRS-2002 [61].

Bian et al. [25] also assessed the consequences of IBD regarding the functional status of patients with the aid of IBD-DI. They found that moderate to severe disability was higher in patients with sarcopenia (48.68 vs. 31.43%, *p* = 0.043), patient-generated SGA ≥ 4 (39.47 vs. 17.14, *p* = 0.003), and high CRP levels (27.63 vs. 11.43%, *p* = 0.021) than in the without-to-minimal disability group [25]. IBD-DI was strongly correlated with WHODAS (r = 0.73, *p* ˂ 0.001) [62]. Leong et al. concluded that IBD can be associated with a high grade of disability, activity limitation, and restriction in participation and correlates IBD-DI with CDAI, partial Mayo Score for UC, and IBD quality-of-life (IBDQ) and concluded that IBD-DI significantly and positively correlates with the IBDQ (r = 0.865, *p* < 0.001), and inversely correlates with CDAI and pMayo. Like Leong, Yoon also correlated IBD-DI with disease activity and HQOL, but more than that, he said that disability was also associated with drug compliance [63,64].

Ciocîrlan et al. [24] assessed malnutrition in recently diagnosed, at least 6 months IBD patients. Malnutrition was defined as a loss of more than 5% of the patient’s initial weight 3 months before registration. Of 625 patients, they reported that 36.3% had malnutrition and that it was more frequent in CD than in UC patients (41.1% vs. 32.4%, *p* = 0.0031). Higher CRP serum values were reported in UC malnourished patients (72 ± 123 mg/dL vs. 34.8 ± 92.8 mg/dL, *p* = 0.035), while serum albumin and hemoglobin values were lower in both UC (3.6 ± 0.9 g/dl vs. 3.9 ± 0.8 g/dL, *p* = 0.032; 11.9 ± 1.9 g/dL vs. 12.6 ± 2.2 g/dL, *p* = 0.006) and CD patients, respectively, (3.6 ± 0.0 g/dL vs. 3.9 ± 0.6 g/dL, *p* = 0.021; 11.7 ± 2.2 g/dL vs. 13.4 ± 1.7 g/dL, *p* = 0.001) [24]. Nguyen et al. found that malnutrition also has a higher prevalence in IBD patients than in the general population, with a similar prevalence between UC and CD, but these findings are more likely due to the UC population being older and with more comorbidities [65].

Higashiyama et al. [32] investigated the effects of frailty and aging on the clinical risk factors of developing elderly-onset UC (EOUC) with the geriatric nutritional risk index (GNRI). They considered that malnutrition is one of the major manifestations of frailty, while Kochar et al. used the Charlson comorbidity index (CCI) to predict the prevalence of frailty in IBD patients [26,32].

Higashiyama et al. [32] divided the patients into four groups according to the GNRI: high risk G3; moderate risk G2; low risk G1; and no risk G0, respectively, with scores ˂82, 82 to ˂92, 92 to ≤98, and ˃98, regarding the risk of EOUC. As age advanced, GNRI decreased, indicating that the higher-risk group is more prevalent with aging. GNRI ≤ 86.82 indicated a strong risk of hospitalization (OR = 4.0, 95% CI 2.5–6.5, *p* ˂ 0.0001) and surgery (OR = 2.7, 95% CI 0.98–7.4) [32].

Kochar et al. [26] investigated the relationship between frailty and mortality in IBD patients. The prevalence of frailty increased with the decade of life 0–9 (6%), 10–19 (2%), 20–29 (4%), 30–39 (5%), 40–49 (7%), 50–59 (6%), 60–69 (7%), 70–79 (9%), 80–89 (12%), ≥90 (25%). Frailty in IBD patients was significantly associated with IBD-related surgery than in fit IBD patients (30% vs. 11% *p* ˂ 0.01). Protein–energy malnutrition (PEM) is the most common ICD code for frailty, with walking difficulty being the second most common frailty associated with the ICD code and unexplained protein–caloric malnutrition being the third most common frailty associated with the ICD code and was discovered in 8% of the frail population. The strongest predictor of frailty was the presence of one or more comorbidities in the CCI (OR 17.31, 95% CI 8.14–36.79). The patients who were frail remained nearly three times more likely to die than those who were fit (aOR 2.90, 95% CI 2.29–3.68) [26].

### 4.4. Limitations and Future Perspective of Our Study

Few studies define sarcopenia in IBD patients, perhaps because the focus is placed more on treating its clinical manifestation, especially the one that involves the gastrointestinal tract. Because sarcopenia is a clinical manifestation present in a large proportion of IBD patients, there is a need to screen patients suffering from IBD for sarcopenia and to prescribe a prompt and complex treatment to limit the clinical and functional impact on the patient’s outcome using a multidisciplinary approach. Moreover, the muscle strength and volume should be assessed with the same instruments to standardize the definition. Because of the great variability in population characteristics, cut-off values should be individualized accordingly.

## 5. Conclusions

In conclusion, sarcopenia is a syndrome that is commonly associated with IBD. There is a high heterogeneity in the modalities of assessment of sarcopenia, and its definition is not yet standardized. One of the main reasons for the lack of homogeneity in definition is the differences in the population’s physical characteristics, activities, and sociocultural characteristics. To provide a correct and comprehensive definition of sarcopenia, it is better to assess both quantity and quality of muscle characteristics using different modalities, such as TPA, TC, CC, waist-to-hip ratio, ASMI, ASM/H^2^, HGS, quadriceps strength, 5CST, 3-m walk test. Sarcopenia and malnutrition are frequently associated with IBD, and they impact the outcome of the disease: disease course and the need for surgery. Therefore, it is important to identify their association because it may have an impact on the prognosis of the disease, taking into consideration that sarcopenia is associated with poor medical and surgical outcomes, together with other risk factors, such as persistent inflammatory markers, NAFLD, malnutrition, frailty, etc. Few studies have analyzed the prognosis relevance of sarcopenia in IBD patients, and there is a need for further studies because IBD is also associated with disability and poor HRQOL, and sarcopenia can increase the grade of disability. The management of sarcopenia is multimodal and requires nutritional, physical, and pharmacological interventions. Every patient with IBD should be assessed to see if sarcopenia is associated and counteract it. The management of patients with sarcopenia and IBD should be made in a multidisciplinary team (gastroenterologist, rehabilitation physician, diabetologist, surgeon) to address the patient as a whole, not only the symptoms of IBD but also the conditions associated with it, in order to offer them a QoL similar to that previous to the ailment. More studies are needed to find the proper physical activity, dietary intake, and medical treatment for IBD patients with sarcopenia.

## Figures and Tables

**Table 1:** The demographic characteristics of the patients.

Author (Reference)	Country	Number of Patients (CD/UC)	Mean Age ± SD	Gender (Female), %	BMI (kg/m^2^)	Sarcopenia Diagnosis (%)	Disease Duration
Subramaniam [20], 2015	Australia	19 (19/0)	33.2 ± 10.7	58	24 ± 4.82	NR	101 ± 99.4 Months
Holt [21], 2017	Australia	34 (34/0)	37.8 ± 14.2	54.5	23.5 ± 4.9	41	5.5 ± 4.0 years
Zhao [22], 2022	China	28 (−/−)	44.1	32.14	21.2	100	NR
Kang [23], 2020	Korea	433(169/264)	39.3	35.9	NR	NR	186.2 months
Ciocîrlan [24], 2019	Romania	625 (241/365)	44.1 ± 14.6	44.05	NR	NR	NR
Bian [25], 2021	China	146 (145/0)	38.89 ± 10.68	33.57	19.67 ± 3.37	44.41	4.99 ± 4.91 years
Kochar [26], 2020	USA	11,001 (−/−)	46	53	NR	NR	NR
Huang [27], 2021	China	72 (48/25)	36.00	28.8	20.13 ± 3.04	NR	5 years
Boparai [28], 2021	India	44 (44/0)	34.4 ± 14.1	36.4	NR	43	48 months
Ge X [29], 2021	China	254 (0/254)	43.9 ± 0.9	36.6	20.8 ± 0.2	50	62.4 ± 4.1 months
Fiorindi [30], 2020	Italy	53 (38/15)	51.08 ± 15.06	4//2	NR	NR	11 years
Zhou [31], 2021	China	122 (122/0)	32.5 ± 12.04	22.13	18.49 ± 2.80	58.85	2.19 ± 3.93 years
Higashiyama [32], 2021	Japan	2778 (0/2778)	NR	NR	NR	NR	NR
Pribadi [33], 2022	Indonesia	85 (37/48)	42	70.06	NR	12.9	NR
Nardone [34], 2022	Switzerland	63 (63/0)	44.2 ± 17.0	46	21.2 ± 3.6	68.3	140.0 ± 106.5 months
Nam [35], 2022	Korea	1027 (854/173)	NR	NR	NR	NR	NR

BMI—body mass index, NR—not reported.

**Table 2:** Cut-off values of skeletal muscle index.

Author (Reference)	Cut-Off Values (cm^2^/m^2^)
Boparai [28], 2021	36.54 ♂
	30.21 ♀
Ge X [29], 2021	42.44 ♂
	33.48 ♀
Zhou [31], 2021	NR
Nardone [34], 2022	52.4 ♂
	38.5 ♀
Nam [35], 2022	49 ♂
	31 ♀

NR—not reported, ♂—males, ♀—females.

**Table 3:** The link between sarcopenia and body composition markers.

Author (Reference)		Patients with Sarcopenia	Patients without Sarcopenia	*p* Value
Boparai [28], 2021 CD patients	Number of patients	19	25	
	BMI (kg/m^2^)	17.2 ± 4.5	21.5 ± 3.4	0.001
	VF area (cm^2^)	77.27 ± 36.9	109.2 ± 58.3	0.04
	SC area (cm^2^)	89.76 ± 65.1	141.7 ± 85.9	0.03
	VF/SC ratio	1.26 ± 0.9	0.87 ± 0.3	0.06
	SMI (cm^2^/m^2^)	27.45 ± 4.9	39.55 ± 4.89	˂0.001
Ge X [29], 2021, UC patients	Number of patients	127	127	
	BMI (kg/m^2^)	20.2 ± 3.5	21.3 ± 3.0	0.006
	VF area (cm^2^)	62.8 ± 40.3	66.4 ± 37.2	0.457
	SC area (cm^2^)	96.2 ± 46.6	115.9 ± 50.4	0.001
	VF/SC ratio	0.75 ± 0.49	0.61 ± 0.31	0.006
	SMI (cm^2^/m^2^)	NR	NR	
Nardone [34], 2022, CD patients	Number of patients	43	20	
	BMI (kg/m^2^)	20.3 ± 3.1	23.3 ± 3.8	0.002
	VF area (cm^2^)	54.0 ± 63.1	63.4 ± 62.7	0.54
	SC area (cm^2^)	94.1 ± 90.4	149.9 ± 84.2	0.009
	VF/SC ratio	0.7 ± 0.6	0.4 ± 0.4	0.04
	SMI (cm^2^/m^2^)	NR	NR	
Nam [35], 2022, CD patients	Number of patients	491	363	
	BMI (kg/m^2^)	20.1 ± 3.3	19.2 ± 3.3	˂0.0001
	VF area (cm^2^)	34.4 ± 28.4	49.7 ± 45.0	˂0.0001
	SC area (cm^2^)	74.0 ± 57.2	93.4 ± 70.9	˂0.0001
	VF/SC ratio	0.68 ± 0.78	0.69 ± 0.58	0.900
	SMI (cm^2^/m^2^)	37.0 ± 7.9	52.5 ± 10.2	˂0.0001
Nam [35], 2022, UC patients	Number of patients	92	81	
	BMI (kg/m^2^)	22.4 ± 3.9	21.6 ± 2.7	˂0.0001
	VF area (cm^2^)	31.6 ± 27.2	42.4 ± 36.8	0.032
	SC area (cm^2^)	63.5 ± 45.6	83.2 ± 57.7	0.015
	VF/SC ratio	0.77 ± 1.06	0.58 ± 0.36	0.108
	SMI (cm^2^/m^2^)	36.9 ± 7.8	53.9 ± 0.6	˂0.0001
		Patients with adverse outcome	Patients without adverse outcome	
Zhou [31], 2021	Number of patients	49	73	
	BMI (kg/m^2^)	17.61 ± 2.41	18.85 ± 2.78	0.012
	VF area (cm^2^)	40.08 ± 42.98	57.54 ± 45.59	0.036
	SC area (cm^2^)	39.18 ± 45.61	62.37 ± 51.44	0.012
	VF/SC ratio	8.06 ± 25.98	1.22 ± 0.99	0.026
	SMI (cm^2^/m^2^)	40.15 ± 6.81	42.93 ± 7.86	0.046

BMI—body mass index; VF—visceral fat SC—subcutaneous fat; SMI—skeletal muscle index.

**Table 4:** Methods to counteract sarcopenia in IBD patients.

**Author (Reference)**	**Method Used**	**Muscle Gain Assessment**	**Muscle Strength Assessment**	**Physical Activity Assessment**
Subramaniam [20]	IFX	Thigh VM	Quadriceps muscle strength at 3 speeds of contraction with an isokinetic dynamometer	IPAQ short form
Zhao [22]	WP RT WP + RT	ASM/H^2^ CC HC Waist/hip ratio	HGS	5CST 3-m walk

IFX—infliximab, VM—muscle volume, IPAQ—International Physical Activity Questionnaire, WP—whey protein, RT—resistance training, ASM/H^2^—height-adjusted appendicular skeletal muscle mass, CC—calf circumference, HC—hip circumference, HGS—hand grip strength, 5CST—5 times chair stand test.

## Data Availability

Not applicable.
